# β-Lactam Adjunctive Therapy Compared to Vancomycin or Daptomycin Monotherapy in Adult Patients With Methicillin-Resistant *Staphylococcus aureus* Bacteremia: An Update Systematic Review, Meta-Analysis, and Trial Sequential Analysis

**DOI:** 10.1155/cjid/3972494

**Published:** 2025-09-04

**Authors:** Changyun Zhao, Wenchao Mao, Difan Lu, Kailun Cai, Changqin Chen, Weihang Hu, Shanmei Lv, Qi Yang

**Affiliations:** ^1^Department of Critical Care Medicine, Zhejiang Hospital, Lingyin Road 12, Hangzhou 310013, Zhejiang, China; ^2^Cardiovascular Ultrasound Center of the First Affiliated Hospital, College of Medicine, Zhejiang University, Hangzhou 310000, Zhejiang, China; ^3^Department of Clinical Laboratory Center, Shaoxing People's Hospital (Shaoxing Hospital), Shaoxing 312000, Zhejiang, China; ^4^Department of Respiratory, The First Hospital of Jiaxing (Affiliated Hospital of Jiaxing University), No. 1882, Nanhu District, Jiaxing 314000, Zhejiang, China

**Keywords:** β-lactams, bacteremia, daptomycin, *methicillin-resistant Staphylococcus aureus*, vancomycin

## Abstract

**Background:** This study evaluated the efficacy and safety of vancomycin (VAN) or daptomycin (DAP) combined with β-lactams (BLs) versus monotherapy (STAN) for *methicillin-resistant Staphylococcus aureus* (MRSA) bacteremia.

**Methods:** PubMed, Web of Science, Embase, and Cochrane Library were searched until September 30, 2024, for RCTs or cohort studies comparing combination therapy (COMBO) and STAN in adult MRSA bacteremia. Outcomes included all-cause mortality, 30-day mortality, clinical failure, and safety. Subgroup and trial sequential analyses were performed.

**Results:** Among 22 studies (3214 patients), the COMBO group did not reduce all-cause mortality (RR = 1.16, 95% CI: 0.91–1.48, *p*=0.24) and 30-day mortality (RR = 1.18, 95% CI: 0.86–1.62, *p*=0.31). Subgroup analyses suggested increased all-cause mortality in high-quality studies (RR = 1.29, 95% CI: 1.00–1.67, *p*=0.05). Additionally, when VAN/DAP was administered randomly, COMBO was associated with higher all-cause mortality (RR = 1.37, 95% CI: 1.05–1.78, *p*=0.02) and 30-day mortality (RR = 1.41, 95% CI: 1.01–1.96, *p*=0.02). However, the COMBO reduced clinical failure rate (RR = 0.78, 95% CI: 0.65–0.93, *p*=0.006), persistent bacteremia (RR = 0.70, 95% CI: 0.54–0.92, *p*=0.01), and relapsed bacteremia (RR = 0.62, 95% CI: 0.48–0.80, *p*=0.0003). No differences were observed in the microbiological failure rate, duration of bacteremia, or length of hospital stay. Furthermore, the COMBO group showed no significant increase in the incidence of acute kidney injury (AKI).

**Conclusions:** COMBO did not lower mortality in MRSA bacteremia and may increase risk in certain subgroups. However, it improved microbiological outcomes without raising AKI risk. However, these microbiological advantages must be weighed against two concerning findings: a nonsignificant trend toward increased Clostridium difficile infection (CDI) risk and elevated mortality signals in high-quality subgroup analyses. Given conflicting mortality signals, cautious clinical application and further RCTs are needed.

## 1. Introduction

*Methicillin-resistant Staphylococcus aureus* (MRSA) is a significant contributor to various medical conditions, including bacteremia, endocarditis, skin and soft tissue infections, as well as bone and joint infections, in addition to being a common cause of hospital-acquired infections [1]. When compared to *methicillin-sensitive Staphylococcus aureus* (MSSA), MRSA is associated with a higher mortality rate in cases of bacteremia, prolonged duration of hospitalization, increased rates of readmission, and elevated overall medical expenses [2, 3]. Consequently, it is essential to effectively manage and treat bacteremia resulting from MRSA. The initial step in the management of MRSA bacteremia involves the identification and eradication of the infection source. Concurrently, the selection and administration of suitable and effective antibiotics are essential for the treatment of MRSA bacteremia [4].

Vancomycin (VAN) remains the preferred therapeutic option for the majority of patients diagnosed with MRSA bacteremia. However, several factors contribute to suboptimal efficacy and a significant rate of clinical failure. These factors include the need for blood drug concentration monitoring, the effect of VAN minimum inhibitory concentration (MIC) values, and the influence of renal function on VAN blood concentration [5–8]. At the same time, the emergence of VAN-resistant strains, including *heterogeneous Vancomycin intermediate–resistant Staphylococcus aureus* (hVISA) and *Vancomycin intermediate–resistant Staphylococcus aureus* (VISA), has raised persistent concerns regarding the effectiveness of VAN [9]. Daptomycin (DAP) serves as an alternative to VAN, particularly in cases where VAN exhibits a high MIC [10]. Recent meta-analysis indicated that DAP treatment for MRSA bacteremia was linked to reduced mortality rate in comparison to VAN. Furthermore, an early transition from VAN to DAP has been significantly correlated with decreased mortality rates [11]. However, it is worth noting that while DAP demonstrates efficacy against the majority of MRSA strains, there have been documented cases of DAP-resistant MRSA, along with a reported clinical failure rate [12–14].

Several new antibiotics are being evaluated for treating MRSA, including ceftaroline (CPT), ceftobiprole, and glycopeptides like dalbavancin, oritavancin, and telavancin, as well as new oxazolidinone such as atezolid. However, there is currently insufficient evidence to demonstrate that these new drugs are more effective than VAN [15]. Despite MRSA's resistance to most β-lactams (BLs), numerous in vitro and in vivo studies have shown that VAN and DAP could work synergistically with different BLs, such as piperacillin/tazobactam and ceftaroline. This combination appears to offer promising new options for treating MRSA bacteremia in clinical settings [16]. This occurrence is referred to as the “seesaw” effect, in which early studies have found that MRSA's sensitivity to BLs improves when glycopeptides or lipopeptides are present [17, 18]. More recent studies have shown that while standard BLs are generally ineffective against MRSA, they can prevent the emergence of glycopeptide-insensitive strains caused by various gene mutations [19, 20].

In recent years, there has been a growing number of studies assessing the effectiveness and safety of VAN or DAP in combination with BLs for treating MRSA bacteremia, and systematic reviews and meta-analyses have been conducted to summarize the research results [21–23]. However, the role of BLs as an adjunct therapy for MRSA bacteremia is still debated, as previous meta-analyses have shown inconsistent results [21–23]. Consequently, we have decided to incorporate recent clinical studies to more accurately evaluate the clinical efficacy and safety of using adjunctive BLs in MRSA bacteremia treatment, and explore the different effects of different antibiotic combinations (e.g., VAN + BLs vs. DAP + BLs) on the results through detailed subgroup analysis, while conducting a trial sequential analysis (TSA) to verify the robustness of the conclusions. These findings provide contemporary evidence-based medical data to inform clinical decision-making regarding optimal antibiotic combination therapies.

## 2. Methods

### 2.1. Protocol and Guidance

This meta-analysis was performed in strict adherence to the Preferred Reporting Items for Systematic Reviews and Meta-Analysis (PRISMA). The protocol of this study was registered in PROSPERO (Registration Number: CRD42024559860).

### 2.2. Literature Search Strategy

We systematically searched four databases from the inception of the database to September 30, 2024, including Web of Science, EMBASE, PubMed, and Cochrane Library. The search was restricted to studies involving human subjects, with no limitations on language. In the process of performing a literature search within PubMed, the methodologies employed for retrieval encompass the use of Medical Subject Headings (MeSH) terms, liberal term retrieval, and Boolean logic operations. Conversely, when executing literature searches in databases such as EMBASE, Web of Science, and the Cochrane Library, we utilized keywords as the primary search strategy. The following search terms were used: (Daptomycin OR Vancomycin) AND (beta-lactams OR cephalosporine OR ceftaroline OR cefazolin) AND (*methicillin-resistant Staphylococcus aureus* OR MRSA) AND (bacteremia OR septicemia OR bloodstream infection). Furthermore, to ensure a thorough identification of all relevant studies, we performed a manual search of recent reviews and the reference lists of pertinent primary studies. Two independent authors (WCM and DFL) executed the search in accordance with the established inclusion and exclusion criteria. Any disagreements regarding the retrieved literature were addressed through discussion. In instances where consensus could not be reached, a third author (CYZ) was involved to render a final decision.

### 2.3. Eligibility Criteria

To assess whether the literature satisfies the eligibility criteria, two authors conducted an independent evaluation of the titles, abstracts, and full texts of the studies. The research screening was performed in accordance with the PICOS principles as follows: (1) Participant: Adult patients diagnosed with bacteremia attributed to MRSA. A pathogen is classified as MRSA if drug sensitivity testing indicates nonsusceptibility to methicillin. (2) Intervention: The COMBO group received treatment with BLs in conjunction with either VAN or DAP. (3) Comparison: The STAN group was administered monotherapy with either VAN or DAP. (4) Outcome: The studies must report at least one relevant outcome. (5) Research type: Eligible studies include randomized controlled trials (RCTs) and cohort studies. Eligible letters, comments, and conference abstracts containing pertinent analytical data will be systematically incorporated into this study to ensure comprehensive evidence synthesis.

The exclusion criteria encompass case-control studies, animal studies, case reports, reviews, systematic reviews, meta-analyses, studies with incomplete raw data, and duplicate publications.

The primary outcome of our meta-analysis was all-cause mortality. In instances where mortality rates were reported at multiple time points, the primary analysis documented mortality at the most recent time point within the study. The secondary outcomes include the following: (1) 30-day mortality (28-day mortality data were not pooled with 30-day mortality); (2) 90-day mortality; (3) clinical failure rate: clinical failure was defined as a composite endpoint that primarily including the 30-day mortality rate, the 60-day mortality rate, persistent bacteremia, and inadequate treatment efficacy necessitating a modification in the type of antibiotic used or the addition of other antibiotics; (4) microbiological failure rate; (5) relapsed bacteremia: Following the previously negative blood culture, the blood culture retaken at least 72 h later again showed positive results; (6) persistent bacteremia: persistent bacteremia (≥ 7 day or ≥ 5 day); (7) duration of bacteremia; (8) length of inpatient admission.

Adverse events mainly include acute kidney injury (AKI), thrombocytopenia, Clostridium difficile infection (CDI), and elevated creatinine phosphokinase (CPK). AKI is defined as an increase in serum creatinine (SCr) of ≥ 0.3 mg/dL (≥ 26.5 μmol/L) within 48 h, or an increase in serum creatinine to ≥ 1.5 times baseline, known or presumed to have occurred within the past 7 days, or a urine volume of < 0.5 mL/kg/h, lasting for 6 h [24].

### 2.4. Data Extraction

The data for this study were independently extracted by two authors (DFL and WCM). Each author performed a comprehensive review of articles that potentially satisfied the inclusion criteria and employed a custom-designed data extraction table to gather pertinent information. In the event of any discrepancies, a third author (CYZ) will be consulted to facilitate discussion and achieve a consensus. The following information will be extracted from each study: (1) Basic research information: including the name of the first author, year of publication, research country, and research type. (2) Baseline characteristics of patients: specifically the number of patients in both the COMBO group and the STAN group. (3) Intervention: encompassing the administration of antibiotics in both the COMBO and STAN groups, including the use of VAN or DAP in the STAN group, the specific BLs utilized in COMBO group, and the dosages of antibiotics administered. (4) Outcomes: the primary outcome is all-cause mortality, and the secondary outcomes are 30-day mortality, 90-day mortality, clinical failure rate, microbiological failure rate, relapsed bacteremia, persistent bacteremia, duration of bacteremia, length of inpatient admission. And the Adverse events include AKI, thrombocytopenia, CDI, and elevated CPK.

### 2.5. Methodological Quality Assessment

The Newcastle Ottawa Scale (NOS) [25] was used to evaluate the quality of observational studies. The NOS checklist comprises three quality parameters: selected populations, groups comparability, and assessment of exposure or results of interest in case-control or cohort studies. The scoring range for each study is from 0 to 9. Studies with a score of ≥ 6 are considered moderate to high quality, otherwise they are deemed to be of low quality. For RCTs, follow the recommendations of the Cochrane Intervention Systems Review manual and use Review Manager 5.4 software [26]. The risks of bias assessment include random sequence generation, allocation concealment, subject and personnel blinding, outcome evaluation blinding, incomplete outcome data, and selective reporting. If all evaluation items are determined to have a low risk of bias, the study is considered to have a low risk of bias. Otherwise, the study is considered to have a high risk of bias.

### 2.6. Statistical Analysis

Data analysis was performed using Review Manager 5.4 software provided by Cochrane International Cooperation organization and STATA version 17.0 (StataCorp, College Station, TX). The significance level for the two-sided test is 0.05, with *p* < 0.05 considered statistically significant. The analysis of metrological data involved mean deviation (MD) and standard deviation (SD), while counting data were analyzed using relative risk (RR) and 95% confidence interval (95% CI). Considering that the studies we included were mainly retrospective cohort studies, and there was significant heterogeneity between studies, we used the DerSimonian-Laird random effect model to synthesize the data. The subgroup analysis plan is as follows: a. Based on the risk of bias (moderate to high-quality or low-quality); b. Subgroup analysis by study type (RCT or Cohort study); c. Subgroup analysis by type of antibiotic used in the standard treatment group (VAN or DAP or VAN/DAP); d. Subgroup analysis by type of BLs used (CPT and other BLs). A funnel plot is used to analyze the potential publication bias, with asymmetry in the plot being evaluated through Egger's test and Begg's test. In cases of identified publication bias, the “trim and fill” method is adopted to incorporate potentially missing studies.

A TSA (version 0.9.5.10 beta) was conducted on the results to mitigate the potential for random errors associated with inadequate sample sizes and repeated testing. The TSA facilitates the calculation of optimal statistical measures and establishes appropriate significance thresholds for meta-analyses. When the cumulative Z curve intersects the TSA significance boundary, enters the area of invalidity, or reaches the predetermined optimal sample size, definitive conclusions can be drawn. Conversely, if the cumulative Z curve does not intersect any boundary, no definitive conclusions can be established. In this TSA, an alpha level of 0.05 (two-tailed) and a beta level of 0.20 were employed to determine the optimal sample size, with the RR reduction calculated based on a 16% decrease in the control group.

## 3. Results

### 3.1. Literature Retrieval

**Figure 1:**
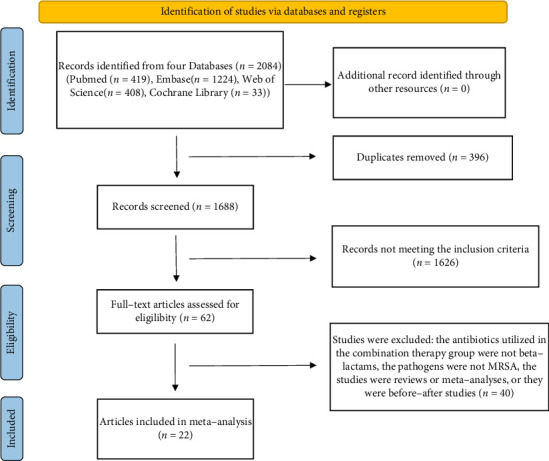
PRISMA flow diagram of the study selection process.

A total of 2084 articles were initially retrieved, including 419 from PubMed, 1224 from EMBASE, 408 from Web of Science, and 33 from the Cochrane Library. Following the removal of duplicate articles, 1688 articles remained. Upon reviewing the titles and abstracts, several articles were excluded, including systematic reviews, case reports, and comments. Ultimately, 62 articles were selected for comprehensive reading. Of these, 40 studies were excluded for various reasons: the antibiotics used in the COMBO group were not BLs, the pathogens were not MRSA, the studies were classified as reviews or meta-analyses, or they were categorized as before–after studies. Consequently, 22 studies [25–46] fulfilled all selection criteria. The detailed PRISMA flowchart depicting the study selection process is provided in Figure 1.

### 3.2. Characteristics of the Included Trials

Table 1 delineates the characteristics of the studies included in the meta-analysis. The studies originated from five different countries and regions, with a significant majority from the United States. Out of the 22 studies included [27–48], three were RCTs [27–29], while the remaining 19 were retrospective cohort studies [30–48]. Within the COMBO group, eight studies utilized VAN [27, 30–36], eight studies employed DAP [28, 37–43], and the remaining six studies utilized either VAN or DAP [29, 44–48]. Regarding the combined administration of BLs, CPT was featured in 10 studies [28, 38–40, 42, 43, 45–48], whereas flucloxacillin, cloxacillin, and other BLs were included in the remaining 12 studies [27, 29–37, 41, 44]. For the primary and secondary outcomes, 18 studies addressed all-cause mortality [27–29, 31–35, 38–42, 44–48], 12 studies focused on 30-day mortality [28, 31–35, 39–41, 44, 47, 48], 4 studies focused on 90-day mortality [27–29, 42], 12 studies reported data on clinical failure rate [28, 29, 31–34, 36, 37, 41–44], and 4 studies reported data on microbiological failure rate [29, 30, 34, 35]. Additionally, 10 studies assessed the duration of bacteremia [27, 28, 31, 34, 35, 40, 42, 44, 45, 48], while 16 studies reported data on relapsed bacteremia [27, 29, 31–35, 38–42, 44–46, 48] and 10 studies on persistent bacteremia [27, 29, 31–35, 41, 44, 48]. The length of inpatient admission was reported in 7 studies [28, 31, 34, 39, 41, 44, 48]. Regarding adverse events, 13 studies provided data on AKI [28–32, 34, 35, 41, 42, 44, 45, 47, 48], 5 studies reported on CDI [35, 41, 42, 44, 47], 5 studies examined elevated CPK levels [28, 40–42, 48], and 3 studies addressed thrombocytopenia [30, 34, 44].

### 3.3. Results of Methodological Quality Evaluation

Upon conducting a thorough evaluation of the methodological quality of the 22 studies included in this analysis, it was determined that two out of three RCTs exhibited a high risk of bias [27, 28], while one RCT was assessed to have a low risk of bias [29]. Among the 19 retrospective cohort studies, 13 trials [30–32, 34, 35, 37, 39–42, 44, 45, 48] were categorized as exhibiting moderate to high quality, whereas the remaining 6 trials [33, 36, 38, 43, 46, 47] were deemed to possess low quality. A comprehensive assessment of the methodological quality for each study is presented in Table 2, along with additional details in Supporting Figures S1 and S2.

### 3.4. Results of the Meta-Analysis and TSA

#### 3.4.1. Primary Outcomes: All-Cause Mortality

**Figure 2:**
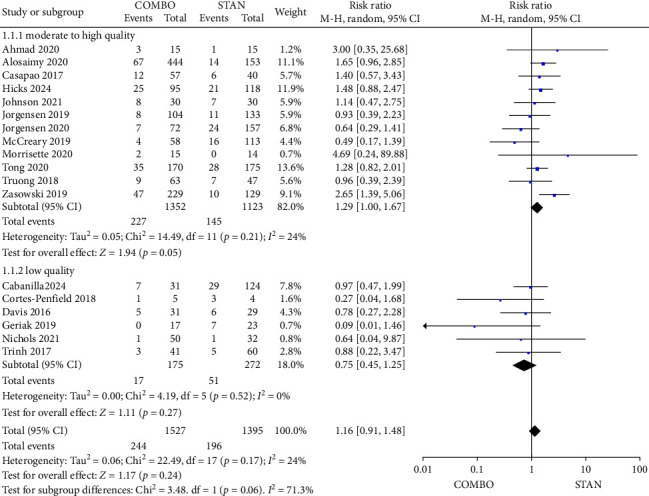
Forest plot comparing the all-cause mortality in the COMBO group to that of in the STAN group.

A meta-analysis including 18 studies [27–29, 31–35, 38–42, 44–48], which included a total of 2922 patients (comprising 1527 patients in the COMBO group and 1395 patients in the STAN group). The random effects model was used for the meta-analysis. The results revealed no statistically significant difference in all-cause mortality between the COMBO and STAN groups (RR = 1.16, 95% CI: 0.91–1.48, *p*=0.24; *I*^2^ = 24%) (Figure 2). In the subgroup analysis, following the exclusion of low-quality studies, the results of the meta-analysis remained statistically insignificant (RR = 1.29, 95% CI: 1.00–1.67, *p*=0.05; *I*^2^ = 24%) (Figure 2). Furthermore, in additional subgroup analyses, irrespective of whether the COMBO group utilized combination therapy with CPT or other BL antibiotics, there was no significant reduction in all-cause mortality observed (Figure S3). The comparison between the COMBO group and the STAN group revealed no statistically significant differences in all-cause mortality across both RCTs and cohort studies (Figure S4). Additionally, the all-cause mortality in the COMBO group did not exhibit a significant decrease, regardless of the administration of VAN or DAP. Notably, when VAN or DAP was administered randomly, the all-cause mortality rate in the COMBO group was unexpectedly higher (RR = 1.37, 95% CI: 1.05–1.78, *p*=0.02; *I*^2^ = 0%) (Figure S5). Given the ambiguity surrounding the specific antibiotic utilized and the heterogeneity among the studies, it is not possible to offer additional clarification regarding this outcome.

**Figure 3:**
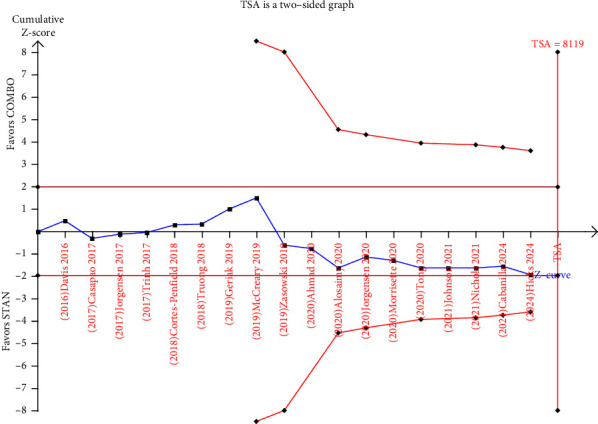
Trial sequential analysis (TSA) for all-cause mortality.

In the course of conducting a TSA, six trials [27, 28, 38, 40, 42, 45] were ignored in interim looks due to too low information used (less than 1%). The results of the TSA indicated that a total of 2922 patients were included in the analysis, while the required information size (RIS) was determined to be 8119. The cumulative Z value did not cross any boundary and the cumulative sample size did not reach the expected sample size. These findings suggest that the meta-analysis may yield false negative conclusions, necessitating the inclusion of additional trials to validate the effect on all-cause mortality (Figure 3).

#### 3.4.2. The Secondary Outcomes

##### 3.4.2.1. 30-Day Mortality

**Figure 4:**
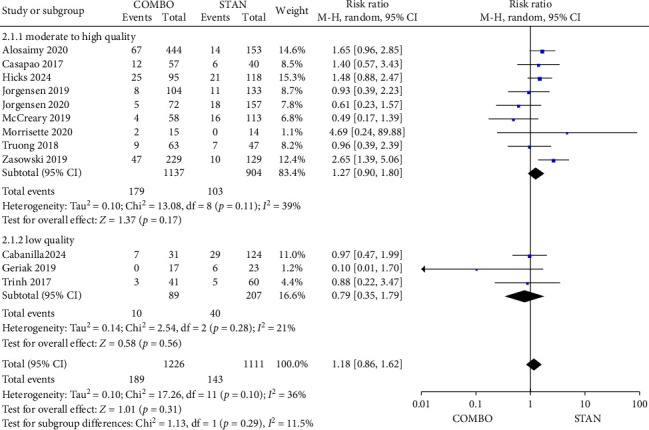
Forest plot comparing the 30-day mortality in the COMBO group to that in the STAN group.

The analysis encompassed 12 studies [28, 31–35, 39–41, 44, 47, 48], which collectively included 2337 patients, with 1226 assigned to the COMBO group and 1111 to the STAN group. These studies provided data regarding 30-day mortality. The findings indicated no statistically significant difference in the 30-day mortality between the two groups (RR = 1.18, 95% CI: 0.86–1.62, *p*=0.31; *I*^2^ = 36%) (Figure 4). In the subgroup analysis, it was observed that when VAN or DAP was administered randomly, the COMBO group exhibited a higher 30-day mortality (RR = 1.41, 95% CI: 1.01–1.96, *p*=0.04; *I*^2^ = 0%) (Figure S6), which aligns with the findings related to all-cause mortality. However, no statistically significant difference in 30-day mortality was identified between the two groups in the other subgroup analyses (Figures S7 and S8).

##### 3.4.2.2. 90-Day Mortality

**Figure 5:**
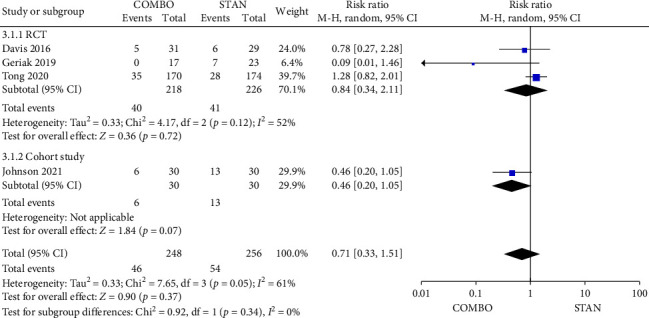
Forest plot comparing the 90-day mortality in the COMBO group to that in the STAN group.

In the analysis of 90-day mortality, only four studies [27–29, 42] provided relevant data. A total of 504 patients were included in the studies, with 248 in the COMBO group and 256 in the STAN group. The findings from the meta-analysis indicated that the COMBO group did not demonstrate a significant reduction in 90-day mortality when compared to the STAN group (RR = 0.71, 95% CI: 0.33–1.51, *p*=0.37; *I*^2^ = 61%) (Figure 5).

##### 3.4.2.3. Clinical Failure Rate

**Figure 6:**
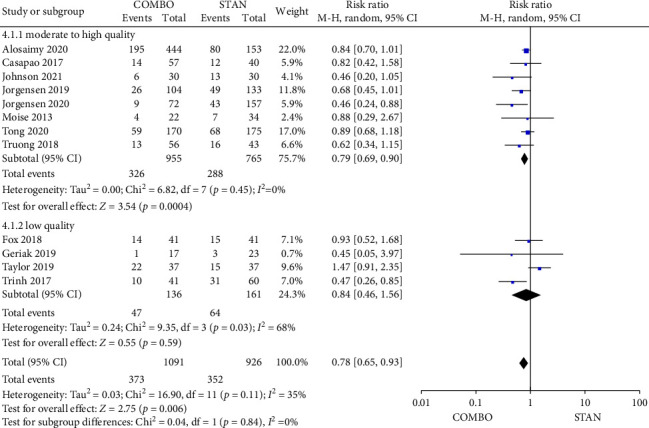
Forest plot comparing the clinical failure rate in the COMBO group to that in the STAN group.

Twelve studies provided data regarding the clinical failure rate [28, 29, 31–34, 36, 37, 41–44], including a total of 2017 patients, with 1091 patients in the COMBO group and 926 patients in the STAN group. The findings from the meta-analysis indicated that the COMBO group was associated with a statistically significant reduction in the risk of clinical failure (RR = 0.78, 95% CI: 0.65–0.93, *p*=0.006; *I*^2^ = 35%) (Figure 6). The results of the subgroup analysis indicated that, following the exclusion of four low-quality studies and the inclusion of eight moderate- to high-quality studies, the outcome of the meta-analysis remained consistent, the COMBO group continued to demonstrate a lower clinical failure rate (RR = 0.79, 95% CI: 0.69–0.90, *p*=0.0004; *I*^2^ = 0%) (Figure 6). Additionally, other subgroups revealed that when VAN or DAP was administered randomly, the administration of DAP with BLs, as well as the COMBO group utilized combination therapy with other BLs, were associated with a reduced risk of clinical failure rate. And no significant differences were found in other subgroups (Figures S9, S10, and S11).

##### 3.4.2.4. Microbiological Failure Rate

**Figure 7:**
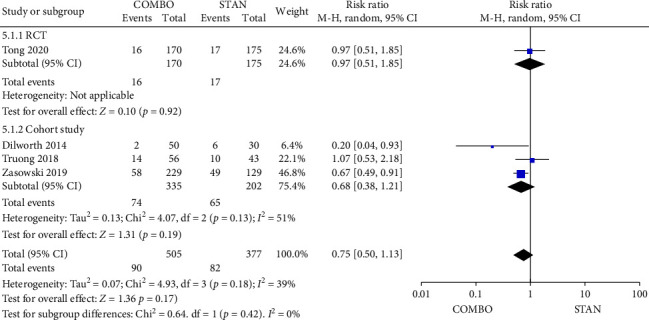
Forest plot comparing the microbiological failure rate in the COMBO group to that in the STAN group.

Four studies provided pertinent data regarding the microbiological failure rate [29, 30, 34, 35], including a total of 882 patients, with 505 patients in the COMBO group and 377 patients in the STAN group. The findings from the meta-analysis indicated that there was no statistically significant difference in the microbiological failure rate between the two groups (RR = 0.75, 95% CI: 0.50–1.13, *p*=0.17; *I*^2^ = 39%) (Figure 7).

##### 3.4.2.5. Persistent Bacteremia

**Figure 8:**
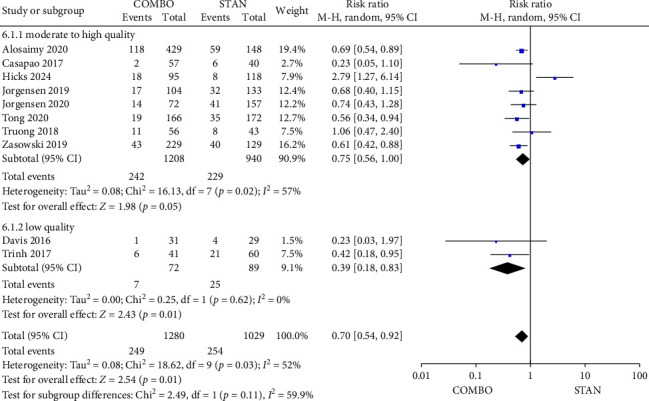
Forest plot comparing the persistent bacteremia in the COMBO group to that in the STAN group.

A meta-analysis of 10 studies [27, 29, 31–35, 41, 44, 48] involving 2309 patients (including 1280 patients in the COMBO group and 1029 patients in the STAN group) showed that the COMBO group could significantly reduce the risk of persistent bacteremia (RR = 0.70, 95% CI: 0.54–0.92, *p*=0.01; *I*^2^ = 52%) (Figure 8). In our subgroup analysis, we observed that the COMBO group demonstrated a reduction in the risk of persistent bacteremia within the moderate to high-quality studies subgroup, the RCT subgroup, and the subgroup involving the combination of VAN with BLs. Furthermore, the concurrent administration of BLs, whether in the form of CPT or other BLs, was associated with a decreased risk of persistent bacteremia (Figures S12, S13, and S14).

##### 3.4.2.6. Duration of Bacteremia

**Figure 9:**
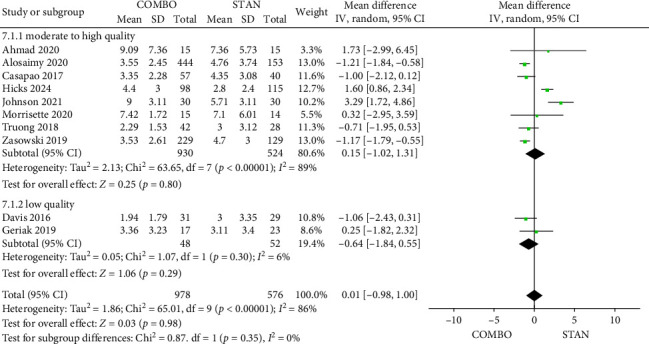
Forest plot comparing the duration of bacteremia in the COMBO group to that in the STAN group.

A total of 10 studies [27, 28, 31, 34, 35, 40, 42, 44, 45, 48] provided data for analysis regarding the duration of bacteremia, including 1554 patients, with 978 patients in the COMBO group and 576 patients in the STAN group. The findings from the meta-analysis indicated that the COMBO group did not significantly reduce the duration of bacteremia when compared to the STAN group (MD = 0.01, 95% CI: −0.98 to 1.00, *p*=0.98; *I*^2^ = 86%) (Figure 9). However, subgroup analyses revealed that the COMBO group was effective in shortening the duration of bacteremia specifically in cases where VAN was combined with BLs, as well as in instances involving when VAN or DAP combined with other BLs, excluding CPT (Figures S15 and S16). Notably, when CPT was utilized in conjunction with VAN or DAP, there was a tendency to prolong the duration of bacteremia (MD = 1.67, 95% CI: 0.61 to 2.74, *p*=0.002; *I*^2^ = 39%) (Figure S16). And no significant differences were found in other subgroups (Figure S17).

##### 3.4.2.7. Relapsed Bacteremia

**Figure 10:**
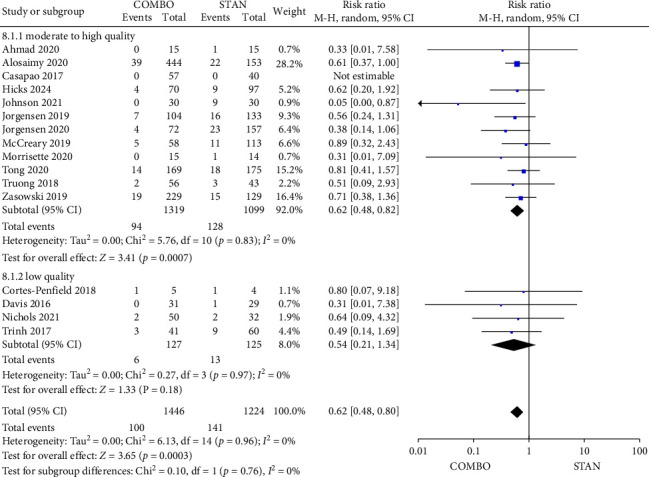
Forest plot comparing the relapsed bacteremia in the COMBO group to that in the STAN group.

A total of 16 studies provided data on relapsed bacteremia [27, 29, 31–35, 38–42, 44–46, 48], encompassing 2670 patients, with 1446 patients in the COMBO group and 1224 patients in the STAN group. The findings from the meta-analysis indicated that the COMBO group was associated with a significant reduction in the risk of relapsed bacteremia (RR = 0.62, 95% CI: 0.48–0.80, *p*=0.0003; *I*^2^ = 0%) (Figure 10). The subgroup analysis indicated that in the moderate- to high-quality studies subgroup, the cohort study subgroup, the subgroup receiving VAN in conjunction with BLs, and the COMBO group demonstrated a significant reduction in the risk of relapsed bacteremia (Figures 10, S18, and S19). Conversely, in the RCTs subgroup and the subgroup employing combination therapy with CPT, no statistically significant difference was observed in the risk of relapsed bacteremia between the two groups (Figures S18 and S20).

##### 3.4.2.8. Length of Inpatient Admission

**Figure 11:**
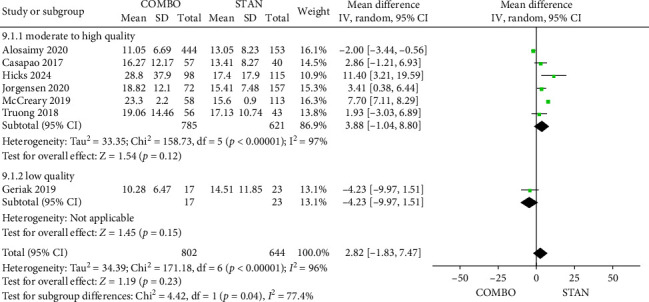
Forest plot comparing the length of inpatient admission in the COMBO group to that in the STAN group.

A total of seven studies [28, 31, 34, 39, 41, 44, 48] involving 1446 patients provided data concerning the length of inpatient admission, comprising 802 patients in the COMBO group and 644 patients in the STAN group. The results of the meta-analysis indicated that there was no statistically significant difference in the length of inpatient admission between the two groups (MD = 2.82, 95% CI: −1.83 to 7.47, *p*=0.23; *I*^2^ = 96%) (Figure 11). Furthermore, no statistically significant differences were observed in other subgroups conducted between the two groups (Figures S21, S22, and S23).

#### 3.4.3. Adverse Events: AKI, Thrombocytopenia, CDI, Elevated CPK

**Figure 12:**
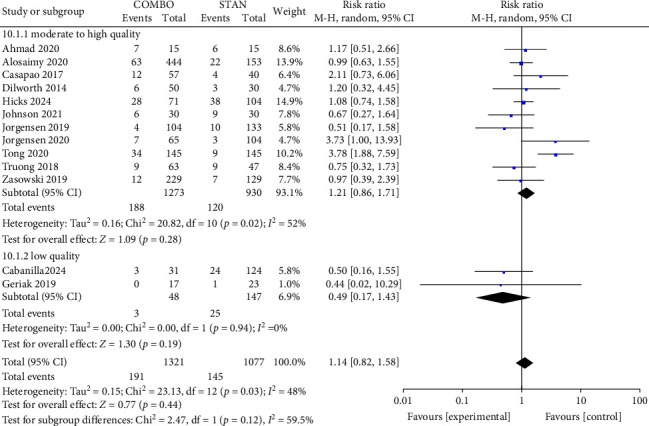
Forest plot comparing the AKI in the COMBO group to that in the STAN.

In the analysis of adverse events, a total of 13 studies [28–32, 34, 35, 41, 42, 44, 45, 47, 48] provided data on AKI, including 2398 patients, with 1321 patients in the COMBO group and 1077 patients in the STAN group. The findings from the meta-analysis indicated that COMBO therapy did not significantly elevate the risk of AKI (RR = 1.14, 95% CI: 0.82–1.58, *p*=0.44; *I*^2^ = 48%) (Figure 12). Furthermore, upon the exclusion of studies deemed to be of low quality, as indicated by NOS scores of less than 6, the meta-analysis results remained unchanged (RR = 1.21, 95% CI: 0.86–1.71, *p*=0.28; *I*^2^ = 52%) (Figure 12). The outcomes of various subgroups were consistent with those of the overall meta-analysis (Figures S24, S25, and S26).

**Figure 13:**
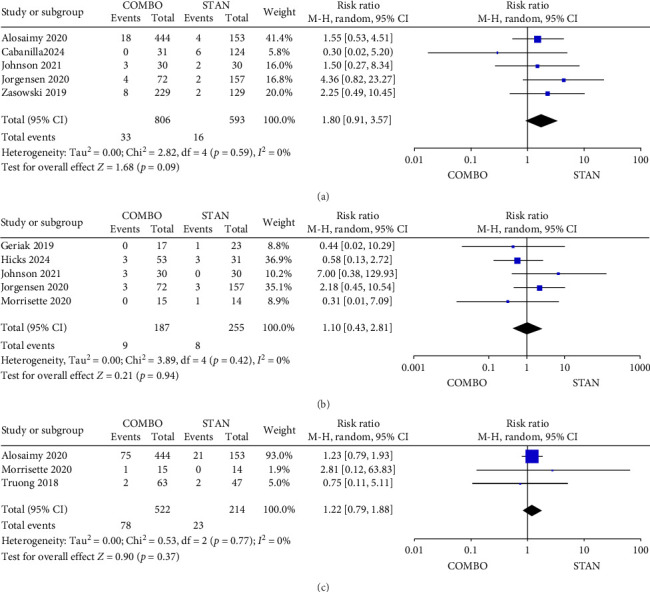
Forest plot comparing the other adverse events in the COMBO group to that in the STAN group ((a) CDI, (b) CPK elevation, (c) thrombocytopenia).

No statistically significant differences were observed between the COMBO group and the STAN group regarding other adverse events, including CDI (RR = 1.80, 95% CI: 0.91–3.57, *p*=0.09; *I*^2^ = 0%) (Figure 13(a)), CPK elevation (RR = 1.1, 95% CI: 0.43–2.81, *p*=0.84; *I*^2^ = 0%) (Figure 13(b)), and thrombocytopenia (RR = 1.22, 95% CI: 0.79–1.88, *p*=0.37; *I*^2^ = 0%) (Figure 13(c)).

### 3.5. Sensitivity Analysis and Publication Bias

**Figure 14:**
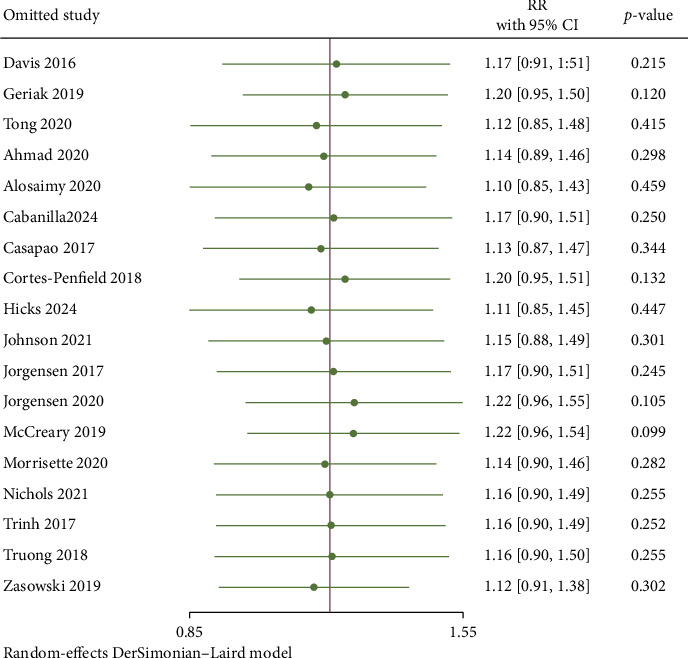
Leave-one-out sensitivity analysis for the all-cause mortality.

**Figure 15:**
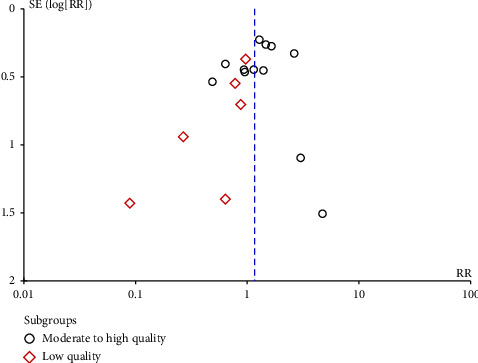
Funnel plot of publication bias analysis included in all-cause mortality analysis literature.

The summary results and subgroup analyses results of the primary outcomes, secondary outcomes, and adverse events of this meta-analysis can be found in Table 3. We performed a leave-one-out sensitivity analysis to assess the robustness of our meta-analysis. In the analysis of all-cause mortality, the exclusion of any individual study did not alter our findings, and there was no statistically significant difference observed between the two groups. This finding suggests that the results of our meta-analysis are robust (Figure 14). We conducted an evaluation of publication bias concerning all-cause mortality utilizing a funnel plot. In the funnel plot, we chose RR as the abscissa and standard error as the ordinate. The funnel plot was visually asymmetrical (Figure 15), suggesting the presence of potential publication bias. The results of Egger's test (*Z* = −1.83, *p*=0.0667) and Begg's test (*Z* = −0.93, *p*=0.3633) indicated that there is no publication bias.

## 4. Discussion

As far as we know, this meta-analysis includes the greatest number of studies and the largest sample size to assess the efficacy and safety of VAN/DAP combined with BLs in the treatment of MRSA bacteremia. While previous similar meta-analyses have been conducted, we have incorporated the most recent trials and updated the current evidence. Our meta-analysis findings showed that there was no significant difference in the risk of all-cause mortality, 30-day mortality, or 90-day mortality between the COMBO group and the STAN group. Additionally, we did not identify any statistically significant subgroup analyses indicating a reduced risk of mortality, when subgroup analysis restricted to high-quality studies (after excluding those with high risk of bias) revealed that combination therapy may increase the risk of all-cause mortality. Moreover, in patients randomized to VAN/DAP, the COMBO regimen was associated with elevated all-cause mortality, including 30-day mortality. However, regarding secondary outcomes, the COMBO group demonstrated better results than the STAN group in decreasing clinical failure, persistent bacteremia, and relapsed bacteremia, as well as in reducing the duration of bacteremia. In our subgroup analysis, we discovered that VAN + BLs were more effective in decreasing persistent bacteremia, relapsed bacteremia, and the duration of bacteremia. On the other hand, DAP + BLs showed superior performance in lowering clinical failure rate. Additionally, there were no significant statistical differences between the two groups concerning adverse events, such as AKI, CDI, CPK elevation, and thrombocytopenia. Neither subgroup analysis showed that the COMBO group had a higher incidence of AKI than the STAN group.

In vitro models have substantiated the efficacy of combination therapy in the management of MRSA bacteremia, demonstrating promising potential. The “seesaw effect” indicates that diminished sensitivity to VAN results in decreased transcription of the mecA gene (responsible for *S. aureus* resistance to oxacillin), thereby enhancing MRSA's susceptibility to BLs [49]. Conversely, the alterations induced by BLs in at least three major cell envelope phenotypes (surface charge, membrane fluidity, and cardiolipin content) may provide a foundation for DAP to enhance its activity against MRSA [50]. Furthermore, in vitro studies have revealed that the combination of cefazolin and DAP significantly lowered the concentration of DAP required to reach 95% growth inhibition (IC_95_) [51].

While in vitro models have indicated the efficacy of combination therapy, our meta-analysis results have not substantiated this finding effectively. When considering all-cause mortality, as well as 30-day and 90-day mortality rates, the COMBO group did not exhibit a statistically significant reduction. Comparable research outcomes have been reported in previous meta-analyses [21–23, 52], which demonstrated that, regardless of the number of studies included, the combination of VAN/DAP and BLs, or the administration of VAN in conjunction with certain BLs (such as ceftaroline) for therapy, did not result in a statistically significant reduction in all-cause mortality, nor in mortality rates at 28, 30, 60, or 90 days within the COMBO group.

While the COMBO group did not exhibit a significant reduction in all-cause mortality overall, potential advantages regarding mortality were identified in subgroup analyses from prior meta-analyses [21, 22]. Specifically, a meta-analysis encompassing 2594 patients across 15 studies concluded that the COMBO group did not significantly decrease all-cause mortality. However, a subgroup analysis focusing on DAP + BLs indicated that the COMBO group was associated with a lower rate of all-cause mortality (RR = 0.53, 95% CI: 0.28–0.98, *p*=0.04) [21]. Another meta-analysis encompassing 13 studies with a total of 1796 patients was conducted. While the COMBO group did not demonstrate significant advantages in terms of all-cause mortality or 30-day mortality, a subgroup analysis revealed that the combination of DAP and CPT was associated with a reduced 30-day mortality rate [22]. In our subgroup analysis, we observed that DAP + BLs appeared to decrease all-cause mortality; however, this finding did not reach statistical significance (RR = 0.65, *p*=0.15). The inclusion of a greater number of studies in our subgroup analysis (6 studies compared to 3 studies) may account for discrepancies in the results, potentially attributable to variations in sample sizes among the studies analyzed. Regarding which type of BLs should be chosen as part of combination therapy, CPT is theoretically an optimal option due to its efficacy against MRSA. Nevertheless, the result of recent meta-analyses [21, 23], including subgroup analyses from our study, indicated that the combination of VAN/DAP with CPT may lower the risk of all-cause mortality; however, the differences observed have not achieved statistical significance. Consequently, the potential advantages of this combination therapy remain ambiguous at this time.

In our subgroup analysis, we observed a noteworthy finding indicating that the COMBO group exhibited a higher all-cause mortality rate when either VAN or DAP was selected without any restrictions (RR = 1.37, 95% CI: 1.05–1.78, *p*=0.02). A thorough examination of the studies incorporated in the subgroup analysis revealed that the research conducted by Sara Alosaimy et al. exerted a considerable influence on our meta-analysis result [44]. This retrospective study comprised a COMBO group of 444 patients and a STAN group of 153 patients, highlighting a notable disparity in sample sizes between the two cohorts. The findings indicated that the COMBO group exhibited a higher all-cause mortality rate; however, this difference did not achieve statistical significance. Upon meticulous review of the literature, it was observed that patients in the COMBO group presented with greater disease severity, as evidenced by a higher APACHE II score (19.1 ± 9.2) in comparison to the STAN group (13.7 ± 7.2). It can be understood that more serious illnesses can lead to higher mortality rate. In another large RCT (CAMERA2) [29], the combination of VAN/DAP with BLs did not demonstrate a reduction in all-cause mortality among patients with MRSA bacteremia when compared to the monotherapy of VAN/DAP. A post hoc analysis of the CAMERA2 trial, using a desirability of outcome ranking approach [53], indicated that combination therapy for MRSA may be associated with poorer clinical outcomes compared to standard therapy. However, this finding did not achieve statistical significance, and the potential advantages of combination therapy cannot be dismissed. This raises an important question: does combination therapy genuinely improve survival rates for patients compared to standard treatment? Currently, the answer remains uncertain, necessitating further large-scale RCTS to elucidate the effects of combination therapy on all-cause mortality in patients with MRSA bacteremia.

In terms of secondary outcomes, the COMBO group demonstrated superior performance in decreasing clinical failure rate, persistent bacteremia, and relapsed bacteremia, in addition to shortening the duration of bacteremia. These findings align with the results of prior meta-analyses [21–23, 52]. In the subgroup analysis, it was observed that the combination of VAN and BLs demonstrated greater efficacy in mitigating the risk of persistent and relapsed bacteremia, as well as in reducing the duration of bacteremia. This result is also similar to the previous meta-analysis result [21]. Additionally, the combination of DAP and BLs was associated with a lower rate of clinical failure. Previous evidence summaries and meta-analyses [54, 55] have demonstrated that DAP exhibited superior efficacy compared to VAN in the treatment of MRSA bacteremia, including lower mortality rate, higher clinical cure rate, and higher microbiological cure rate. The intricacy of this matter has been heightened by the incorporation of BLs in therapeutic regimens. In the absence of a direct comparative analysis between VAN and DAP, it remains inconclusive whether the combination of DAP with BLs demonstrates greater efficacy than the combination of VAN with BLs. As a BL antibiotic exhibiting activity against MRSA, it is hypothesized that the clinical efficacy of CPT would be enhanced when administered in conjunction with VAN or DAP for the treatment of MRSA infections. Nevertheless, our subgroup analysis revealed that the combination of VAN/DAP with CPT was associated with a prolonged duration of bacteremia when compared to the STAN group. Upon examining the relevant literature, it was determined that the primary factor contributing to this outcome was the variability in the severity of patients within the COMBO and STAN groups. Specifically, individuals in the COMBO group tend to exhibit a greater degree of disease severity, as evidenced by a higher proportion of patients presenting with “SBP < 90 mmHg or requiring vasopressor support” in the study conducted by Hicks et al. [48]. This finding suggested that the patients in the COMBO group were in a more critical condition. Furthermore, the research by Johnson et al. indicated that patients in the COMBO group had already experienced standard treatment failure prior to the initiation of combination therapy [42]. Given the increased severity of the patient's condition, it is reasonable to expect a prolonged duration of bacteremia. However, this has impacted the interpretation of the results of our meta-analysis.

Notably, we observed a clinically important dissociation between outcomes in the COMBO group: while microbiological and treatment success metrics improved, the mortality rate did not decrease. This apparent paradox may reflect several factors that warrant careful consideration. This finding aligns with previous reports and may be explained by several key factors: (1) Baseline disease severity: As demonstrated by Alosaimy et al. [44], COMBO-treated patients had significantly higher APACHE II scores, and Hicks et al. [48] reported a greater proportion of hypotensive patients in the COMBO group. (2) Treatment timing: some of included studies utilized COMBO therapy as salvage treatment rather than initial treatments. (3) Therapeutic heterogeneity: Variability in antibiotic dosing, duration differences, timing of combination initiation. These factors collectively suggest that the mortality outcomes may reflect patient population characteristics and treatment variability rather than therapeutic inefficacy.

From the perspective of safety, the combination of VAN/DAP and BLs seemed to be secure and did not substantially elevate the risks associated with AKI, CDI, CPK elevation, or thrombocytopenia. The subgroup analysis pertaining to AKI did not reveal any evidence suggesting that any specific combination, including the combination of VAN and BLs, would markedly elevated the risk of AKI. Notably, our subgroup analysis restricted to high-quality studies revealed substantial heterogeneity (*I*^2^ = 52%) in AKI outcomes. This moderate-to-high heterogeneity likely stems from three key sources: (1) variability in AKI diagnostic criteria across studies (particularly between KDIGO and RIFLE classifications), (2) differences in baseline renal function among enrolled populations, and (3) variation in concomitant nephrotoxic medication use. These factors underscore the importance of cautious interpretation when applying these findings to clinical practice. We have highlighted the nonsignificant but clinically important trend toward increased CDI risk in the COMBO group (RR = 1.80, 95% CI: 0.91–3.57, *p*=0.09) as a potential safety signal, While not statistically significant, the 80% relative increase in CDI risk warrants consideration when prescribing combination therapy to high-risk patients. In the context of combination therapy involving BLs, particularly the VAN + BLs regimen, it is crucial to minimize exposure to nephrotoxic agents due to the nephrotoxic potential of VAN. Furthermore, the possibility of additional adverse effects should prompt careful consideration of patient selection before clinical application of combination therapy.

Despite conducting a systematic review and meta-analysis of the available literature, the heterogeneity among the studies constrained the interpretation of outcomes. Most of the studies primarily focused on combination therapy during the early stages of treatment; however, some incorporated salvage therapy to assess clinical efficacy. Additionally, various factors such as drug dosage, treatment duration, timing of administration, infection site, source control, and pathogen resistance can significantly influence the outcomes. The meta-analysis indicated potential advantages of combining VAN/DAP with BLs, a critical unresolved question remains regarding which patient populations derive the most benefit from increased use of BLs, as well as which specific BLs are most appropriate for use. Addressing these questions necessitates large-scale RCTs and pertinent subgroup analyses.

Our meta-analysis has the following advantages. First, we systematically evaluated the efficacy and safety of VAN/DAP combined with BLs in the treatment of MRSA bacteremia. To date, this meta-analysis encompasses the greatest number of included studies and the largest sample size. Notably, several outcome measures—particularly AKI (*I*^2^ = 52%) and duration of bacteremia (*I*^2^ = 89%)—demonstrated moderate to substantial heterogeneity in our analysis. These findings suggest that the results for these particular outcomes should be interpreted with caution. We conducted sensitivity analysis and TSA to assess the reliability of our findings. Second, we conducted several meaningful subgroup analyses to evaluate the differences in efficacy and safety of different antibiotic combinations in treating MRSA bacteremia. However, our meta-analysis has several limitations. First, most of the included studies were retrospective, with only three being prospective. Second, the sample sizes varied among the included studies, leading to significant heterogeneity. Although we used a random effects model to reinforce the results, caution is still needed when interpreting our findings. Third, there exist notable variations in the timing, dosage, and duration of antibiotic administration across various studies, which present a considerable challenge in the interpretation of our research findings. Fourth, certain studies exhibit a significant risk of bias, which substantially undermines the credibility of the conclusions drawn from meta-analyses. Fifth, while several included studies reported outcomes as medians with interquartile ranges (IQRs), we converted these to MD and SD using the validated estimation methods of Wan et al. (2014) [56] and Luo et al. (2018) [57] to enable meta-analysis. However, we acknowledge this approach carries important limitations: These conversion methods presume underlying normal distributions; however, these data are likely to exhibit non-normal distribution, which could introduce systematic bias.

## 5. Conclusion

In conclusion, our meta-analysis suggest that, in comparison to STAN treatment, COMBO treatment was associated with a decreased risk of clinical failure, persistent bacteremia, and relapsed bacteremia, as well as in reducing the duration of bacteremia. However, it did not appear to affect all-cause mortality and may increase risk in certain subgroups. Furthermore, COMBO treatment did not seem to increase the risk of adverse events, including AKI, CPK elevation, and thrombocytopenia, but there was a trend toward increased CDI risk with COMBO, this potential safety signal warrants particular consideration. Future research should prioritize RCTs that investigate the specific aspects of combination therapy, including combination therapy combinations, doses, administration methods, and duration of treatment, to further assess the evidence regarding its impact on efficacy and safety.

## Figures and Tables

**Table 1:** The detailed characteristics of the included studies.

Trial	Location	Study design	Group	No. of patients	Intervention	All-cause mortality	28-day mortality	30-day mortality	90-day mortality	Clinical failure rate	Microbiological failure rate	Duration of bacteremia (day)	Relapsed bacteremia	Persistent bacteremia	Adverse events	Length of inpatient admission
Davis 2016	Australia	RCT	STAN	29	VAN 1.5g bid	6/29	5/29		6/29			3.0 ± 3.35	1/29	4/29		
			COMBO	31	VAN 1.5g bid + flucloxacillin 2g q6h for the first 7 days after randomization	5/31	5/31		5/31			1.94 ± 1.79	0/31	1/31		

Geriak 2019	America	RCT	STAN	23	VAN or DAP 6–8 mg/kg	7/23		6/23	7/23	3/23		3 (1, 5.3)			AKI (1/23) Asymptomatic elevated CPK (1/23) Eosinophilic pneumonia (0/23)	12 (8, 23)
			COMBO	17	DAP 6–8 mg/kg + CPT 0.6g q8h	0/17		0/17	0/17	1/17		3 (1.5, 5.5)			AKI (0/17) Asymptomatic elevated CPK (0/17) Eosinophilic pneumonia (1/17)	11 (6, 14)

Tong 2020	Australia, Singapore, Israel, New Zealand	RCT	STAN	178	VAN or DAP 6–10 mg/kg	28/174			28/174	68/175	17/175		18/175	35/172	AKI (9/145)	
			COMBO	174	VAN or DAP 6–10 mg/kg + (flucloxacillin 2g q6h/cloxacillin 2g q6h/cefazolin 2g q8h)	35/170			35/170	59/170	16/170		14/169	19/166	AKI (34/145)	

Dilworth 2014	America	Retrospective cohort study	STAN	30	VAN						6/30				Nephrotoxicity (3/30)	
			COMBO	50	VAN + β-Lactams						2/50				Nephrotoxicity (6/50)	

Casapao 2017	America	Retrospective cohort study	STAN	40	VAN	6/40		6/40		12/40		4 (2.5, 6.5)	0/40	6/40	Nephrotoxicity (4/40)	13.5 (8, 18.75)
			COMBO	57	VAN + β-Lactams	12/57		12/57		14/57		3 (2, 5)	0/57	2/57	Nephrotoxicity (12/57)	14.5 (9, 25)

Jorgensen 2019	America	Retrospective cohort study	STAN	133	VAN	11/133		11/133		49/133			16/133	32/133	Nephrotoxicity (10/133)	
			COMBO	104	VAN + CFZ	8/104		8/104		26/104			7/104	17/104	Nephrotoxicity (4/104)	

Trinh 2017	America	Retrospective cohort study	STAN	60	VAN	5/60		5/60		31/60			9/60	21/60		
			COMBO	41	VAN + CFZ	3/41		3/41		10/41			3/41	6/41		

Truong 2018	America	Retrospective cohort study	STAN	47	VAN	7/47		7/47		16/43	10/43	3 (1,5)	3/43	8/43	AKI (9/47) thrombocytopenia (2/47)	15 (11, 25)
			COMBO	63	VAN + β-Lactams	9/63		9/63		13/56	14/56	3 (1,3)	2/56	11/56	AKI (9/63) thrombocytopenia (2/63)	18 (10, 29)

Zasowski 2019	America	Retrospective cohort study	STAN	129	VAN	10/129		10/129			49/129	4 (3,7)	15/129	40/129	Nephrotoxicity (7/129) CDI (2/129)	
			COMBO	229	VAN + Cefepime	47/229		47/229			58/229	3 (2,5.5)	19/229	43/229	Nephrotoxicity (12/229) CDI (8/229)	

Taylor 2019	America	Retrospective cohort study	STAN	37	VAN					15/37						
			COMBO	37	VAN + β-Lactams					22/37						

Moise 2013	America	Retrospective cohort study	STAN	34	DAP					7/34						
			COMBO	22	DAP + β-Lactams					4/22						

Cortes-Penfield 2018	America	Retrospective cohort study	STAN	5	DAP	3/4							1/4			
			COMBO	4	DAP + CPT	1/5							1/5			

McCreary 2019	America	Retrospective cohort study	STAN	113	DAP	16/113		16/113					11/113			15.6 ± 0.9
			COMBO	58	DAP + CPT	4/58		4/58					5/58			23.3 ± 2.2

Morrisette 2020	America	Retrospective cohort study	STAN	14	DAP	0/14		0/14				6.7 (3.6,10.9)	1/14		Asymptomatic elevated CPK (1/14) thrombocytopenia (0/14)	
			COMBO	15	DAP + CPT	2/15		2/15				7.6 (6.3,8.4)	0/15		Asymptomatic elevated CPK (0/15) thrombocytopenia (1/15)	

Jorgensen 2020	America	Retrospective cohort study	STAN	157	DAP	24/157		18/157		43/157			23/157	41/157	AKI (3/104) CDI (2/157) Asymptomatic elevated CPK (7/157)	14 (11, 21)
			COMBO	72	DAP + β-Lactams	7/72		5/72		9/72			4/72	14/72	AKI (7/65) CDI (4/72) Asymptomatic elevated CPK (3/72)	16 (12, 28)

Johnson 2021	America	Retrospective cohort study	STAN	30	DAP	7/30			7/30	13/30		5 (4,8)	9/30		CDI (2/30) AKI (9/30) Asymptomatic elevated CPK (0/30)	
			COMBO	30	DAP + CPT	8/30			8/30	6/30		9 (7,11)	0/30		CDI (3/30) AKI (6/30) Asymptomatic elevated CPK (3/30)	

Fox 2018	America	Retrospective cohort study	STAN	41	VAN					15/41						
			COMBO	41	DAP + CPT					14/41						

Alosaimy 2020	America	Retrospective cohort study	STAN	153	DAP/VAN	14/153		14/153		80/153		4.2 (2.5, 7.5)	22/153	59/153	Nephrotoxicity (22/153) thrombocytopenia (21/153) CDI (4/153)	12 (8,19)
			COMBO	444	DAP/VAN + β-Lactams	67/444		67/444		195/444		3.3 (2.0, 5.3)	39/444	118/444	Nephrotoxicity (63/444) thrombocytopenia (75/444) CDI (18/444)	10 (7,16)

Ahmad 2020	America	Retrospective cohort study	STAN	15	DAP/VAN	1/15						7 (4, 11)	1/15		AKI (6/15)	
			COMBO	15	DAP/VAN + CPT	3/15						8 (5, 14)	0/15		AKI (7/15)	

Nichols 2021	America	Retrospective cohort study	STAN	56	DAP/VAN	1/32							2/32			
			COMBO	66	DAP/VAN + CPT	1/50							2/50			

Cabanilla 2024	America	Retrospective cohort study	STAN	124	DAP/VAN	29/124		29/124							AKI (24/124) CDI (6/124) Neutropenia (9/124)	
			COMBO	31	DAP/VAN + CPT	7/31		7/31							AKI (3/31) CDI (0/31) Neutropenia (0/31)	

Hicks 2024	America	Retrospective cohort study	STAN	115	DAP/VAN	21/118		21/118				2.8 ± 2.4	9/97	8/118	AKI (38/104) Neutropenia (9/89) Asymptomatic elevated CPK (3/31)	17.4 ± 17.9
			COMBO	98	DAP/VAN + CPT	25/95		25/95				4.4 ± 3	4/70	18/95	AKI (28/71) Neutropenia (6/78) Asymptomatic elevated CPK (3/53)	28.8 ± 37.9

*Note:* STAN, standard therapy; COMBO, standard therapy combined with β-lactams; VAN, vancomycin; DAP, daptomycin; CPT, ceftaroline; CPK, creatinine phosphokinase; CFZ, cefazolin.

Abbreviations: AKI, acute kidney injury; CDI, Clostridium difficile infection; RCT, Randomized controlled trial.

**Table 2:** Methodological quality assessment of the included studies.

Trial	Quality evaluation	Case definition	Representativeness	Selection of controls	Definition of controls	Comparability	Ascertainment of exposure	Same method?	Non-response rate
Dilworth 2014	6	1	1	0	1	1	0	1	1
Casapao 2017	7	1	1	1	1	1	0	1	1
Jorgensen 2019	6	1	1	1	1	0	0	1	1
Trinh 2017	4	1	1	0	0	0	0	1	1
Truong 2018	7	1	1	0	1	1	1	1	1
Zasowski 2019	7	1	1	1	1	0	1	1	1
Taylor 2019	5	1	1	0	1	0	1	1	0
Moise 2013	7	1	1	1	1	2	0	1	0
Cortes-Penfield 2018	5	1	1	0	1	0	1	1	0
McCreary 2019	6	1	1	1	1	1	0	1	0
Morrisette 2020	7	1	1	0	1	2	0	1	1
Jorgensen 2020	6	1	1	1	1	1	0	1	0
Johnson 2021	6	1	1	0	1	1	0	1	1
Fox 2018	4	1	1	0	1	0	0	1	0
Alosaimy 2020	7	1	1	1	1	0	1	1	1
Ahmad 2020	6	1	1	0	1	1	1	1	0
Nichols 2021	4	1	1	0	0	1	0	1	0
Cabanilla 2024	5	1	1	0	1	1	0	1	0
Hicks 2024	6	1	1	0	1	1	0	1	1

**Table 3:** Summary of meta-analysis results.

Outcomes	Number of studies	RR or MD (95% CI)	*p*	*I* ^2^
**Primary outcome**				
All-cause mortality	18	**1.16 [0.91, 1.48]**	**0.24**	24%
Low risk of bias	12	1.29 [1.00, 1.67]	0.05	24%
High risk of bias	6	0.75 [0.45, 1.25]	0.27	0%
RCT	3	0.84 [0.34, 2.11]	0.72	52%
Cohort study	15	1.19 [0.90, 1.56]	0.22	24%
VAN	6	1.28 [0.83, 1.99]	0.26	27%
DAP	6	0.65 [0.36, 1.18]	0.15	23%
VAN/DAP	6	1.37 [1.05, 1.78]	0.02	0%
CPT	9	0.96 [0.60, 1.54]	0.87	29%
Other β-lactams	9	1.26 [0.94, 1.68]	0.12	25%

**Secondly outcomes**				
*30-day mortality*	12	**1.18 [0.86, 1.62]**	**0.31**	36%
Low risk of bias	9	1.27 [0.90, 1.80]	0.17	39%
High risk of bias	3	0.79 [0.35, 1.79]	0.56	21%
RCT	1	0.10 [0.01, 1.70]	0.11	—
Cohort study	11	1.23 [0.91, 1.66]	0.17	29%
VAN	5	1.38 [0.86, 2.22]	0.18	30%
DAP	4	0.56 [0.26, 1.20]	0.14	14%
VAN/DAP	3	1.41 [1.01, 1.96]	0.04	0%
CPT	5	0.94 [0.49, 1.82]	0.85	48%
Other β-lactams	7	1.31 [0.89, 1.91]	0.17	33%

*90-day mortality*	4	**0.71 [0.33, 1.51]**	**0.37**	61%
*Clinical failure rate*	12	**0.78 [0.65, 0.93]**	**0.006**	35%
Low risk of bias	8	0.79 [0.69, 0.90]	0.0004	0%
High risk of bias	4	0.84 [0.46, 1.56]	0.59	68%
RCT	2	0.88 [0.67, 1.16]	0.38	0%
Cohort study	10	0.75 [0.60, 0.94]	0.01	44%
VAN	5	0.76 [0.52, 1.13]	0.18	63%
DAP	5	0.64 [0.45, 0.92]	0.02	0%
VAN/DAP	2	0.86 [0.73, 1.00]	0.05	0%
CPT	3	0.71 [0.44, 1.16]	0.17	4%
Other β-lactams	9	0.78 [0.64, 0.96]	0.02	45%

*Microbiological failure rate*	4	**0.75 [0.50, 1.13]**	**0.17**	39%
*Persistent bacteremia*	10	**0.70 [0.54, 0.92]**	**0.01**	52%
Low risk of bias	8	0.75 [0.56, 1.00]	0.05	57%
High risk of bias	2	0.39 [0.18, 0.83]	0.01	0%
RCT	2	0.54 [0.32, 0.88]	0.01	0%
Cohort study	8	0.74 [0.54, 1.01]	0.06	59%
VAN	6	0.61 [0.47, 0.79]	0.0002	0%
DAP	1	0.74 [0.43, 1.28]	0.28	—
VAN/DAP	3	0.94 [0.46, 1.91]	0.87	84%
CPT	1	2.79 [1.27, 6.14]	0.01	—
Other β-lactams	9	0.65 [0.55, 0.76]	< 0.00001	0%

*Duration of bacteremia*	10	**0.01 [**−**0.98, 1.00]**	**0.98**	86%
Low risk of bias	8	0.15 [−1.02, 1.31]	0.80	89%
High risk of bias	2	−0.64 [−1.84, 0.55]	0.29	6%
RCT	2	−0.64 [−1.84, 0.55]	0.29	6%
Cohort study	8	0.15 [−1.02, 1.31]	0.80	89%
VAN	4	−1.06 [−1.53, −0.60]	< 0.00001	0%
DAP	3	1.49 [−0.78, 3.76]	0.20	68%
VAN/DAP	3	0.44 [−2.01, 2.90]	0.72	94%
CPT	5	1.67 [0.61, 2.74]	0.002	39%
Other β-lactams	5	−1.11 [−1.49, −0.74]	< 0.00001	0%

*Relapsed bacteremia*	16	**0.62 [0.48, 0.80]**	**0.0003**	0%
Low risk of bias	12	0.62 [0.48, 0.82]	0.0007	0%
High risk of bias	4	0.54 [0.21, 1.34]	0.18	0%
RCT	2	0.77 [0.40, 1.48]	0.44	0%
Cohort study	14	0.59 [0.44, 0.78]	0.0003	0%
VAN	6	0.61 [0.39, 0.96]	0.03	0%
DAP	5	0.49 [0.23, 1.04]	0.06	14%
VAN/DAP	5	0.66 [0.46, 0.95]	0.02	0%
CPT	7	0.61 [0.32, 1.14]	0.12	0%
Other β-lactams	9	0.62 [0.47, 0.82]	0.0010	0%

*Length of inpatient admission*	7	**2.82 [**−**1.83, 7.47]**	**0.23**	96%
Low risk of bias	6	3.88 [−1.04, 8.80]	0.12	97%
High risk of bias	1	−4.23 [−9.97, 1.51]	0.15	—
RCT	1	−4.23 [−9.97, 1.51]	0.15	—
Cohort study	6	3.88 [−1.04, 8.80]	0.12	97%
VAN	2	2.49 [−0.66, 5.63]	0.12	0%
DAP	3	2.97 [−2.57, 8.52]	0.29	91%
VAN/DAP	2	4.07 [−9.00, 17.14]	0.54	90%
CPT	3	4.82 [−3.35, 13.00]	0.25	88%
Other β-lactams	4	1.29 [−2.07, 4.64]	0.45	79%

**Adverse events**				
*CDI*	5	**1.80 [0.91, 3.57]**	**0.09**	0%
*CPK elevation*	5	**1.10 [0.43, 2.81]**	**0.84**	0%
*Thrombocytopenia*	3	**1.22 [0.79, 1.88]**	**0.37**	0%
*AKI*	13	**1.14 [0.82, 1.58]**	**0.44**	48%
Low risk of bias	11	1.21 [0.86, 1.71]	0.28	52%
High risk of bias	2	0.49 [0.17, 1.43]	0.19	0%
RCT	2	2.29 [0.39, 13.53]	0.36	41%
Cohort study	11	1.01 [0.81, 1.26]	0.94	1%
VAN	5	0.96 [0.61, 1.51]	0.85	0%
DAP	3	1.22 [0.31, 4.74]	0.78	59%
VAN/DAP	5	1.24 [0.74, 2.09]	0.41	71%
CPT	5	0.96 [0.71, 1.32]	0.82	0%
Other β-lactams	8	1.36 [0.82, 2.25]	0.24	62%

*Note:* Values in bold represent the overall risk ratio (RR) or mean difference (MD) along with their confidence intervals, while values in standard font are used for subgroup analyses.

## Data Availability

Our data came from other clinical studies, and the datasets used and analyzed are available from the corresponding author upon reasonable request.

## Nomenclature

VAN: Vancomycin
DAP: Daptomycin
BLs: β-lactams
MRSA: *Methicillin-resistant Staphylococcus aureus*
RCTs: Randomized controlled trials
COMBO: Combination therapy
STAN: Standard therapy
TSA: Trial Sequential Analysis
AKI: Acute kidney injury
MSSA: *Methicillin-sensitive Staphylococcus aureus*
MIC: Minimal inhibitory concentration
hVISA: Heterogeneous Vancomycin intermediate*–*resistant Staphylococcus aureus
VISA: Vancomycin intermediate–resistant Staphylococcus aureus
CPT: Ceftaroline
PRISMA: Preferred Reporting Items for Systematic Reviews and Meta-Analysis
MeSH: Medical subject heading
CDI: Clostridium difficile infection
CPK: Creatinine phosphokinase
SCr: Serum creatinine
NOS: The Newcastle Ottawa Scale
MD: Mean deviation
SD: Standard deviation
RR: Relative risk
CI: Confidence interval
RIS: Required information size
IC_95_: 95% growth inhibition

## Supporting Information

Figure S1. Methodological quality evaluation of included RCTs in the meta-analysis: risk-of-bias summary.

Figure S2. Methodological quality evaluation of included RCTs in the meta-analysis: risk-of-bias graph.

Figure S3. Subgroup analysis: different effects of CPT and other β-lactams on all-cause mortality.

Figure S4. Subgroup analysis: different effects of RCTs and cohort studies on all-cause mortality.

Figure S5. Subgroup analysis: different effects of VAN, DAP, and DAP/VAN on all-cause mortality.

Figure S6. Subgroup analysis: different effects of VAN, DAP, and DAP/VAN on 30-day mortality.

Figure S7. Subgroup analysis: different effects of RCTs and cohort studies on 30-day mortality.

Figure S8. Subgroup analysis: different effects of CPT and other β-lactams on 30-day mortality.

Figure S9. Subgroup analysis: different effects of VAN, DAP, and DAP/VAN on clinical failure rate.

Figure S10. Subgroup analysis: different effects of CPT and other β-lactams on clinical failure rate.

Figure S11. Subgroup analysis: different effects of RCTs and cohort studies on clinical failure rate.

Figure S12. Subgroup analysis: different effects of VAN, DAP, and DAP/VAN on persistent bacteremia.

Figure S13. Subgroup analysis: different effects of CPT and other β-lactams on persistent bacteremia.

Figure S14. Subgroup analysis: different effects of RCTs and cohort studies on persistent bacteremia.

Figure S15. Subgroup analysis: different effects of VAN, DAP, and DAP/VAN on duration of bacteremia.

Figure S16. Subgroup analysis: different effects of CPT and other β-lactams on duration of bacteremia.

Figure S17. Subgroup analysis: different effects of RCTs and cohort studies on duration of bacteremia.

Figure S18. Subgroup analysis: different effects of RCTs and cohort studies on relapsed bacteremia.

Figure S19. Subgroup analysis: different effects of VAN, DAP, and DAP/VAN on relapsed bacteremia.

Figure S20. Subgroup analysis: different effects of CPT and other β-lactams on relapsed bacteremia.

Figure S21. Subgroup analysis: different effects of VAN, DAP, and DAP/VAN on length of inpatient admission.

Figure S22. Subgroup analysis: different effects of CPT and other β-lactams on length of inpatient admission.

Figure S23. Subgroup analysis: different effects of RCTs and cohort studies on length of inpatient admission.

Figure S24. Subgroup analysis: different effects of VAN, DAP, and DAP/VAN on AKI.

Figure S25. Subgroup analysis: different effects of CPT and other β-lactams on AKI.

Figure S26. Subgroup analysis: different effects of RCTs and cohort studies on AKI.
